# Low 25-Hydroxyvitamin D Levels Are Associated With Residual Dizziness After Successful Treatment of Benign Paroxysmal Positional Vertigo

**DOI:** 10.3389/fneur.2022.915239

**Published:** 2022-06-22

**Authors:** Yunqin Wu, Kun Han, Weiwei Han, Zhenyi Fan, Min Zhou, Xiaoxiong Lu, Xiaoxia Liu, Li Li, Liwen Du

**Affiliations:** ^1^Department of Neurology, Hwa Mei Hospital, University of Chinese Academy of Science, Ningbo, China; ^2^Department of Rehabilitation, Hwa Mei Hospital, University of Chinese Academy of Science, Ningbo, China; ^3^Department of Emergency, Hwa Mei Hospital, University of Chinese Academy of Science, Ningbo, China

**Keywords:** benign paroxysmal positional vertigo, 25-hydroxy vitamin D, residual dizziness, canalith repositioning procedure, risk factor

## Abstract

**Objective:**

Vitamin D (Vit D) regulates calcium and phosphate metabolism and helps to maintain otolith organ function. Residual dizziness (RD) is one of the most common complications after the successful treatment of benign paroxysmal positional vertigo (BPPV). Various theories have been suggested to explain the cause of RD, and otolith organ disorder is the most evident cause of RD. This study aimed to investigate the association between serum levels of Vit D and the occurrence of RD after the successful treatment of BPPV.

**Methods:**

A prospective study including patients who were diagnosed with de novo posterior semicircular canal-type BPPV (PC-BPPV) was conducted at our institution from May 2017 to May 2019. All the patients underwent canalith repositioning procedures and were followed up. Univariate and multivariate analyses were performed to investigate the relationship between serum 25-hydroxy vitamin D (25(OH)D) levels and RD occurrence after successful BPPV treatment.

**Results:**

In total, 123 patients with PC-BPPV were enrolled, and 41.5% (51/123) experienced RD. The serum level of 25(OH)D was significantly lower in PC-BPPV patients with RD [median 16.2 ng/ml (IQR 12.9–22.1)] than in patients without RD [median 20.5 ng/ml (IQR 16.5–26.5)] (*P* = 0.001). In multivariate models comparing the prevalence of RD in the insufficient group [25(OH)D ≥ 20 to <30 ng/ml], deficient group [25(OH)D < 20 ng/ml] and normal group [25(OH)D ≥ 30 ng/ml], the 25(OH)D levels in the deficient group were associated with the occurrence of RD (odds ratio = 5.48, 95% confidence interval = 1.08–27.71; *P* = 0.04).

**Conclusion:**

Low 25(OH)D levels are associated with the development of RD in patients with PC-BPPV after successful treatment. Further efforts to validate and elucidate the mechanism are needed.

## Introduction

Human vestibular dysfunction is a common problem in the clinic. Among vestibular disorders, benign paroxysmal positional vertigo (BPPV) is the most common cause of recurrent vertigo, accounting for ~20% of vestibular complaints (1). This condition is characterized by brief repeated episodes of vertigo that are triggered by changes in head position, and it is often accompanied by nausea and vomiting. It is widely accepted that the pathophysiology of BPPV is caused by dislodged otoconia floating freely into the semicircular canals or adhering to the cupula of the semicircular canals (2). Canalith repositioning procedures (CRPs) can place the dislodged otoconia in the utricle and are effective and simple to perform. Indeed, nearly 90% of patients can be cured after one or two sessions of such treatments (1, 2).

However, 38–61% of patients with BPPV may experience a series of non-specific symptoms, such as dizziness, imbalance, or light-headedness, after the successful treatment of BPPV, which is called residual dizziness (RD). RD not only seriously interferes with daily activities but also results in anxiety, depression and falling, especially in elderly individuals (3). Although the pathogenesis of RD remains unclear, otolith organ dysfunction is the primary pathological factor (4, 5). By measuring cervical and ocular vestibular evoked myogenic potentials (c/oVEMP) in patients with BPPV, the results of previous studies support the above theory (6).

Vitamin D (Vit D) is a fat-soluble vitamin, and its main function is the regulation of calcium and phosphate metabolism. As otoconia in the otolith organ are composed of calcium carbonate and protein, alterations in calcium metabolism may induce changes in the otoconial structure and otolith organ status (7). Therefore, Vit D may be closely associated with the maintenance of otolith organ function. Vit D receptor-mutant mice exhibit a series of vestibular dysfunctions (8). The size of otoconia is increased, whereas the density is decreased in ovariectomized osteopenic/osteoporotic female adult rats (9). The diameter and density of the globular substance in Vit D-deficient mice are increased, but supplementation with Vit D mitigates these changes (10). Similarly, a clinical study reported that 66% of people with Vit D deficiency/insufficiency have abnormal VEMP results (11). Additionally, Vit D deficiency is closely related to BPPV occurrence and recurrence, and Vit D supplementation has beneficial effects in the treatment and prevention of BPPV (12, 13).

Animal experiments and clinical studies have suggested that Vit D-mediated effects on otolith function may be involved in the maintenance of calcium homeostasis in the inner ear. Based on these findings, we hypothesized that low serum Vit D levels may be associated with the development of RD after the successful treatment of BPPV. This study explores and validates this hypothesis.

## Methods

This single-center, prospective study was performed at the Department of Neurology and Emergency, Hwa Mei Hospital, University of Chinese Academy, from May 2017 to May 2019. Approval was obtained from our institutional review board (protocol numbers KY-2017-014 and KY-2020-038). Written informed consent was obtained from all participants and the study was conducted in accordance with the Declaration of Helsinki.

All patients who were admitted to the Department of Neurology and Emergency and complained of acute-onset vertigo or dizziness were recruited. The BPPV diagnosis was based on the presence of recurrent vertigo and characterized nystagmus during provocative maneuvers. Patients who were previously diagnosed with BPPV and had secondary factors, such as head trauma, vestibular neuritis, Meniere's disease, migraines, or history of ear surgery, who received long-term steroid therapy or vitamin D supplementation, and who had parathyroid disease or serious liver and kidney diseases that affect vitamin D levels were excluded. To exclude confounding factors, we only included patients who were diagnosed with unilateral posterior semicircular canal-type BPPV (PC-BPPV).

**Figure 1:**
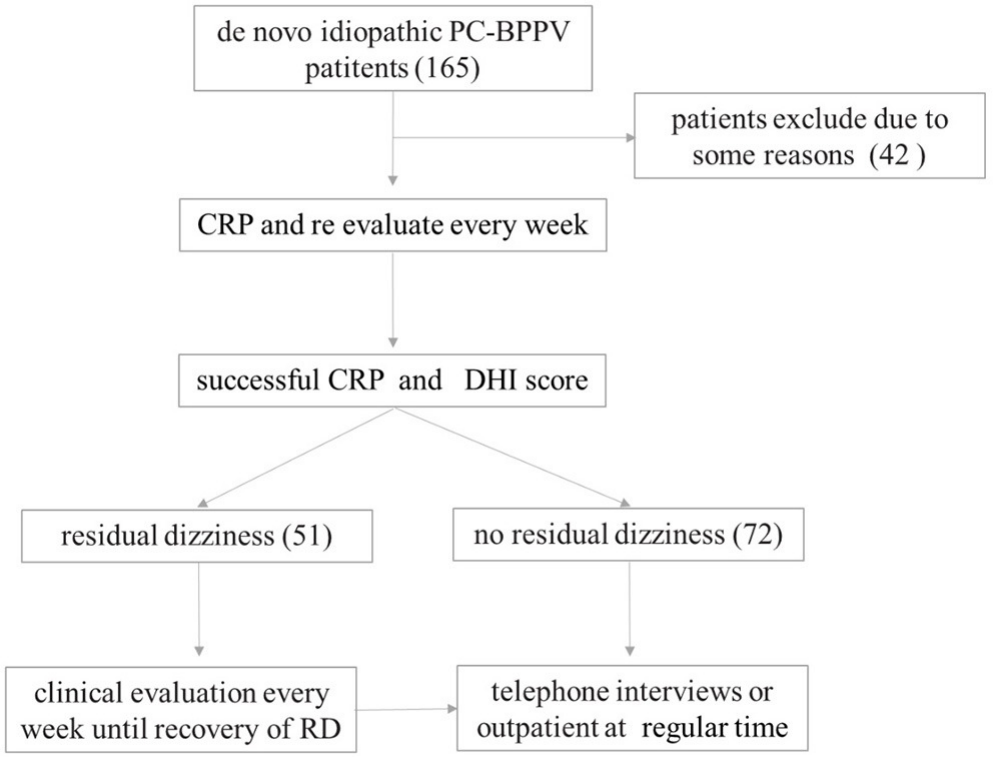
Study flow diagram demonstrating patient enrollment and progress.

Once the BPPV diagnosis was confirmed, patients were treated with appropriate CRP. After execution of the maneuver, the patients were re-evaluated with the same diagnostic session every 7 days; if nystagmus was still present, the CRP was repeated up to a maximum of two times in the same session. Patients who failed to achieve a clinical cure after two sessions of CRP were excluded. Once successful treatment of BPPV was confirmed, patients were assessed for whether they experienced RD and were asked to complete the Chinese version of the Dizziness Handicap Inventory (DHI) (14). We performed a structured interview consisting of two dichotomous questions, to which the only possible answers were “YES” or “NO”. The first question, which aimed to identify the persistent dizziness symptoms, was “Do you feel as unsteady as you were before the onset of BPPV in recent days?”. If the answer was “YES”, we further asked the next question. The second question, which aimed to verify the onset of RD, was “Are these symptoms significantly relieved after CRP?”. If the answer was “No”, the patient was considered to have RD after excluding other disorders using caloric test, pure-tone audiometry, video head impulse test or imaging exam. We recorded data, such as the time of onset and duration, demographics, clinical characteristics, and laboratory indicators. RD was defined as a feeling of unsteadiness and/or light-headedness and/or dizziness in the absence of true vertigo and nystagmus. Patients who complained of RD were evaluated every 7 days until the symptoms disappeared. Then, we performed telephone interviews or outpatient visits at 1, 3, 6 12, and 24 months. The flowchart of the study is presented in Figure 1.

### Measurement of Serum 25-Hydroxyvitamin D

Fasting early-morning venous blood was collected and stored at −80°C for analysis. Serum 25(OH)D was measured using an API3200 liquid chromatography mass spectrometer/mass spectrometer system (Applied Biosystems, Foster City, CA).

### Statistical Analysis

Continuous variables following normal distributions are expressed as the mean ± standard deviation; those that did not follow a normal distribution are expressed as the median and interquartile range (IQR). Categorical variables are presented as the number of patients and percentage. Differences between the two groups were analyzed using a *t*-test, chi-square test, or Mann–Whitney *U*-test. Binary logistic regression analysis was adjusted to evaluate the risk factors for RD in BPPV patients. In addition, multivariate analysis models were used to assess the occurrence of RD after the successful treatment of BPPV according to the international classification of vitamin D (the sufficient group was used as the reference). All the analyses were performed using SPSS (version 22.0, Chicago, IL, USA), and *P* < 0.05 was considered significant.

## Results

### Demographics and Clinical Characteristics of the Participants

A total of 165 patients diagnosed with *de novo* idiopathic PC-BPPV were recruited; 42 patients were eliminated from the study for the following reasons: 11 patients required more than four CRP session; for five patients, the symptoms were not ameliorated after CRP; six patients refused to participate in this study; 13 patients lost to follow-up; and seven patients developed other medical conditions. Ultimately, 123 patients participated in this study: 83 females (67.5%, mean age: 58.01 ± 12.88) and 40 males (32.5%, mean age: 59.6 ± 12.21). For recovery, 70 (56.9%) patients required a single CRP session, 25 (20.3%) patients required two CRP sessions, 18 (14.6%) patients required three CRP sessions, and 10 (8.1%) patients required up to 4 CRP sessions. During follow-up, 49 (39.8%) patients reported one or more episodes of relapse of vertigo. The demographic data are summarized in Table 1.

**Table 1:** Demographical and clinical features of PC-BPPV patients with and without RD.

	**RD**	**NRD**	***P***
Number	51	72	
Age (years)	60.94 ± 13.29	58.50 ± 11.21	0.246
Sex (F/M)	38/13	45/27	0.161
Side (R/L)	24/27	31/41	0.660
BMI (kg/m^2^)	23.3 ± 2.88	23.7 ± 3.32	0.485
Hypertension *n* (%)	20 (39.2%)	24 (33.3%)	0.503
Diabetes mellitus *n* (%)	12 (23.5%)	14 (19.4%)	0.585
Duration of symptoms before CRP (day)	3.0 (1.0–7.0)	3 (1.0–5.0)	−0.401
Number of CRP	1.84 ± 1.04	1.67 ± 0.94	0.311
1	27	43	
2	10	15	
3	9	9	
4	5	5	
DHI scores	28.67 ± 12.05	25.69 ± 9.51	0.110
Recurrence BPPV	2 2(43.1%)	27 (37.5%)	0.529
Albumin (g/l)	42.44 ± 3.81	42.59 ± 3.51	0.294
Creatinine (μmol/l)	55.6 (48.4–68.6)	59.7 (51.65–68.7)	0.105
Blood urea nitrogen (mmol/l)	4.60 (4.07–5.23)	4.64 (3.88–5.62)	0.401
Uric acid (μmol/l)	279.3 [(234.9–317.6) 5.23]	278.4 (249.3–335.0)	0.488
Total cholesterol (mmol/l)	4.56 ± 1.16	4.65 ±0.93	0.133
HDL-C (mmol/l)	1.37 (1.10–1.58)	1.31 (1.06–1.57)	0.606
LDL-C (mmol/l)	2.56 (1.72–3.2)	2.70 (2.08–3.07)	0.558
Triglycerides (mmol/l)	1.23 (0.90–1.78)	1.25 (0.84–1.80)	0.888
25(OH)D (ng/ml)	16.2 (12.9–22.1)	20.5 (16.5–26.5)	0.001

*PC-BPPV, posterior semicircular canal benign paroxysmal positional vertigo; RD, residual dizziness; BMI, body mass index is the weight in kilograms divided by the square of the height in meters; CRP, canalith repositioning procedure; DHI, dizziness handicap inventory; HDL, high-density lipoprotein; LDL, low-density lipoprotein. Values are expressed as n (%), mean ± SD or median (interquartile range). P-values were calculated using an independent samples t-test or the nonparametric Mann-Whitney U-test. P < 0.05 was considered significant*.

### Serum 25(OH)D Levels and the Risk Factors for RD

A total of 123 PC-BPPV patients were included in the study, and 51 patients complained of RD after the successful treatment of BPPV. The results of univariate analysis are displayed in Table 1; the level of serum 25(OH)D in patients with RD was significantly lower than that in patients without RD **[**median 16.2 ng/ml (IQR 12.9–22.1) vs. median 20.5 ng/ml (IQR 16.5–26.5), *P* = 0.001**]**. A multivariate logistic regression model showed that a low 25(OH) D level was a risk factor for RD (*P* = 0.003, odds ratio = 1.11, 95% confidence interval 1.03–1.19) (Table 2). Furthermore, according to the international classification criteria for vitamin D values (15), the prevalence of RD among subgroups ranged from 15.4% (2/13) to 52.2% (35/67). In multivariate analysis comparing the insufficient group and the deficient group with the 25(OH) D normal group, increased risk of experiencing RD after the successful treatment of BPPV associated with the lower 25(OH) D levels in the deficient group (OR 5.48, 95% CI: 1.08–27.71; *P* = 0.04) after adjusting for possible confounders (Table 3).

**Table 2:** Multiple logistic regression analysis to identify independent risk factors for residual dizziness after successful treatment of BPPV.

**Variables**	**OR (95% CI)**	***P***
25(OH)D	1.041–1.186	0.002

*Adjusted for age, sex, duration of symptoms before CRP, number of CRPs, DHI score, albumin, creatinine, and total cholesterol*.

**Table 3:** Multivariate analyses for the occurrence of RD after successful treatment of BPPV according to the 25-hydroxyvitamin D classification criteria.

**25-hydroxyvitamin D**	**RD (*n*/%)**	**Crude OR (95% CI), *P***	**Adjusted OR (95% CI), *P***
Normal (*n* = 13)	2 (15.4%)	Reference	Reference
Insufficiency (*n* =43)	14 (32.5%)	2.66 (0.52–13.64), 0.242	2.61 (0.48–14.23), 0.268
Deficiency (*n* = 67)	35 (52.2%)	6.01 (1.24–29.24), 0.026	5.41 (1.06–27.61), 0.042

*Adjusted for age, sex, BMI, duration of symptoms before CRP, number of CRPs, albumin, creatinine, and total cholesterol*.

## Discussion

Several studies have reported that Vit D is closely related to BPPV; however, no studies have explored serum Vit D levels in BPPV patients with RD. In this study, we compared the serum levels of 25(OH)D in patients with BPPV with or without RD after successful treatment and found that a low level of 25(OH)D may be a risk factor for the development of RD in patients with BPPV after successful treatment.

BPPV is a benign disease; most patients have a good recovery, and vertigo symptoms disappear shortly, even when patients receive no treatment (1, 2). However, some patients report a non-specific sensation of unsteadiness, light-headedness, disorientation, or **drowsiness after** successful treatment. This condition is referred to as RD and can last up to several months. RD is common in patients who have undergone successful treatment of BPPV, with a reported incidence ranging from 38 to 61% due to different inclusion criteria and inconsistencies in the definition of RD duration. Overall, RD results in a significant negative impact on daily function and quality of life (3, 4). Nonetheless, the origin of RD is not yet clear, and no guidelines recommend any drugs for this condition. Therefore, exploring potential risk factors for the occurrence of RD is of great significance for follow-up studies on its pathogenesis and drug development.

Several hypotheses have been proposed to explain the occurrence of RD, such as the persistence of debris in the canal, otolith dysfunction, coexisting vestibular disease, incomplete central nervous system adaptation, mental health, or autonomic dysfunction, but the exact causes are still unclear (3, 5). Among these hypotheses, otolith dysfunction is considered the most persuasive. CRPs can bring dislodged otoconia back to the utricle, but otolith dysfunction remains. Yetiser et al. compared cVEMP parameters among subgroups of patients with BPPV and found that patients with a long period of symptoms had longer p1 latency than those with a shorter duration of symptoms (16). Similarly, a study conducted by Oh et al. reported that in BPPV, an increased cVEMP modified interaural amplitude difference ratio on the affected side was associated with RD after recovery from BPPV (17). According to Seo et al. (18), RD is related to the results of a second oVEMP test performed after successful treatment, suggesting that the origin of RD is persistent utricular dysfunction even after successful CRP.

Otoconia are biocrystals anchored to the macular sensory epithelium of the utricle and saccule for motion sensing and balance, and they undergo degeneration, absorption, and regeneration *in vivo* (19). The capacity of otoconium dissolution depends on the concentration of Ca^2+^ in the endolymph (20). A variety of calcium channel-related proteins are expressed in the epithelia of semicircular canal ducts, and these proteins participate in spatiotemporal modulation of Ca^2+^ levels and maintain the homeostasis of Ca^2+^ levels in the endolymph. Vit D may affect the maintenance of calcium homeostasis *in vivo* by regulating the expression of calcium-related proteins (21, 22). In addition, 1,25-dihydroxyvitamin D3 can significantly upregulate the expression of calcium channel-related proteins, such as transient receptor potential vanilloid 5, calbindin-D9K and calbindin-D28K, in rat semicircular canal epithelial-derived cells (23). Vit D receptor mutant mice display vestibular dysfunctions, and the morphology and density of otoconia change in animal models of vitamin D deficiency. Furthermore, a clinical study found that 66% of people with Vit D deficiency/insufficiency have abnormal VEMP results (8–10). These data suggest that Vit D is important for maintaining vestibular function.

Calcium metabolism is involved in the synthesis and absorption of otoconia, and thus, defects in systemic calcium metabolism may induce changes in the otoconial structure and otolith organ status, leading to otolith organ disorder (18, 24). Some investigations have confirmed that BPPV is closely related to otolith dysfunction (25, 26). The amplitude of oVEMP transiently increases in the affected ear after a successful liberating maneuver, suggesting utricular dysfunction caused by a possible deficit of the matrix that embeds the otoconia on the macula (27). Another study conducted by Von Brevern et al. (28) found that patients with BPPV showed a reduced otolith-ocular reflex and that the change persisted for 1 month after successful treatment of BPPV. Hence, otolith dysfunction can persist and remain in the form of RD after successful BPPV treatment. Suh et al. reported that BPPV patients with osteopenia more frequently suffer from RD after successful treatment of BPPV than those without osteopenia (29). Similarly, Jiang et al. confirmed that a reduction in bone mineral density is independently associated with the occurrence of RD in patients after successful treatment of PC-BPPV (30).

CRPs can move dislodged otoconia to the utricle, but some fragments of otoconia may remain in the semicircular canal. The Vit D endocrine system is crucial for maintaining calcium homeostasis in the endolymph, and disturbances in calcium metabolism may affect the ability of the endolymph to dissolve exfoliated otoconia debris (18, 19). Therefore, we hypothesize that abnormal serum Vit D levels may be closely related to the development of RD. In this study, we enrolled 123 patients with PC-BPPV who had been successfully treated, and 41.5% (51/123) of them experienced RD, which was similar to previous research. To date, no studies have investigated the relationship between serum levels of 25(OH)D and the occurrence of RD. Regardless, we found that the serum level of 25(OH)D in BPPV patients with RD was significantly lower than that in patients without RD, and multivariate analysis suggested that a lower serum level of 25(OH)D was associated with the occurrence of RD in patients with PC-BPPV after successful treatment.

Betahistine is widely used to ameliorate dizziness in patients due to its effects of increasing labyrinthine microcirculation as well as regulating intracellular Ca^2+^ by acting on histamine receptors located in the vestibular periphery (31, 32). However, conflicting data exist regarding the efficacy of betahistine for preventing or treating RD. To date, there is no relevant drug recommendation for the management of the very common RD after successful treatment of BPPV. This study showed that serum 25(OH)D levels in BPPV patients with RD were significantly lower than those in patients without RD, and a lower serum level of 25(OH)D was associated with the occurrence of RD in patients with PC-BPPV after successful treatment. Several reports on vitamin D supplementation and a variety of health outcomes include improved cardiovascular health, bone health, fall and fracture risk, and even colorectal cancer prevention. Similarly, recent studies have found that Vit D supplementation not only reduces the recurrence rate of BPPV but also relieves the intensity of vertigo. Therefore, supplementation with vitamin D and calcium would help restore the healthy condition of otoconia in patients with vitamin D deficiency and may have potential health benefits in the treatment of vestibular disorders. Whether Vit D supplementation can improve RD in patients with BPPV is unclear. It is at least reasonable that Vit D supplementation can improve RD in patients with BPPV who complain of RD after successful treatment, when serum Vit D levels are lower than normal. RD is so common after successful treatment of BPPV that even if the supplementation of vitamin D could relieve symptoms in only a small percentage of cases, many patients will experience improvement.

The present study had a few limitations. First, it was a single-center small sample observational study, which only recruited *de novo* PC-BPPV patients, and whether these findings are consistent with other subtypes or patients with recurrent BPPV is unclear. Second, patients with BPPV did not undergo a systematical vestibular assessment, using caloric test, pure-tone audiometry, video head impulse test or VEMP, the influence of coexisting vestibular diseases such as vestibular neuritis and Meniere's disease on the development of RD cannot be completely excluded. Finally, we did not investigate vestibular function instrumentally, such as with c/o VEMP or caloric tests, during the evaluation period. Therefore, further studies should be carried out to explore the probable mechanism, which may offer new ideas or directions for addressing and coping these symptoms.

## Conclusion

Low 25(OH) D levels are associated with the development of RD after successful treatment of BPPV.

## Data Availability Statement

The raw data supporting the conclusions of this article will be made available upon request to: duliwen1020@163.com.

## Ethics Statement

The studies involving human participants were reviewed and approved by Hwa Mei Hospital, University of Chinese Academy. The patients/participants provided their written informed consent to participate in this study.

## Author Contributions

YW and LD designed and supervised the study. YW wrote the paper. KH, WH, ZF, MZ, XLu, XLiu, and LL collected and preprocessed the data. WH and ZF performed the statistical analyses. All authors contributed to the article and approved the submitted version.

## Funding

This study was supported by Ningbo Medical Key Discipline (Grant No. B12), Ningbo Medical Support Discipline (Grant No. F16), Ningbo Natural Science Foundation (Grant No. 202003N4240), Hwa Mei Foundation (Grant Nos. 2021HMKY 30, 2022HMZY102, and 2020HMKY22), and Zhejiang Medicine and Health Science and Technology Project (2021KY1015).

## Conflict of Interest

The authors declare that the research was conducted in the absence of any commercial or financial relationships that could be construed as a potential conflict of interest.

## Publisher's Note

All claims expressed in this article are solely those of the authors and do not necessarily represent those of their affiliated organizations, or those of the publisher, the editors and the reviewers. Any product that may be evaluated in this article, or claim that may be made by its manufacturer, is not guaranteed or endorsed by the publisher.

## References

[1] Editorial Board of Chinese Journal of Otorhinolaryngology Head and Neck Surgery. Guideline of diagnosis and treatment of benign paroxysmal positional vertigo (in Chinese). Chin J Otorhinolaryngol Head Neck Surg. (2017) 52:173–7. 10.3760/cma.j.issn.1673-0860.2017.03.003 28395487

[2] Kim H Park J Kim J. Update on benign paroxysmal positional vertigo. J Neurol. (2021) 268:1995–2000. 10.1007/s00415-020-10314-7 33231724 PMC7684151

[3] Wu P Yang J Huang X Ma Z Zhang T Li H. Predictors of residual dizziness in patients with benign paroxysmal positional vertigo after successful repositioning: a multi-center prospective cohort study. J Vestib Res. (2021) 31:119–29. 10.3233/VES-201535 33285662

[4] Çetin YS Çagaç A Düzenli U Bozan N. Elasan S. Residual dizziness in elderly patients after benign paroxysmal positionalvertigo. Otorhinolaryngol Relat Spec. (2021) 8:1–8. 10.1159/000516961 34237746

[5] Vaduva C Estéban-Sánchez J Sanz-Fernández R. Martín-Sanz E. Prevalence and management of post-BPPV residual symptoms. Eur Arch Otorhinolaryngol. (2018) 275:1429–37. 10.1007/s00405-018-4980-x 29687182

[6] Fife TD Colebatch JG Kerber KA Brantberg K Strupp M Lee H. Practice guideline: cervical and ocular vestibular evoked myogenic potential testing: report of the Guideline Development, Dissemination, and Implementation Subcommittee of the American Academy of Neurology. Neurology. (2017) 89:2288–96. 10.1212/WNL.0000000000004690 29093067 PMC5705249

[7] Büki B Jünger H Zhang Y Lundberg YW. The price of immune responses and the role of vitamin D in the inner ear. Otol Neurotol. (2019) 40:701–9. 10.1097/MAO.0000000000002258 31194714 PMC6578582

[8] Minasyan A Keisala T Zou J Zhang Y Toppila E Syvala H. Vestibular dysfunction in vitamin D receptor mutant mice. J Steroid Biochem Mol Biol. (2009) 114:161–6. 10.1016/j.jsbmb.2009.01.020 19429446

[9] Vibert D Sans A Kompis M Travo C Muhlbauer R Tschudi I. Ultrastructural changes in otoconia of osteoporotic rats. Audiol Neurootol. (2008) 13:293–301. 10.1159/000124277 18391565

[10] Song P Zhao X Xu Y Zhao Z Liu Y Gao Q. Morphological effect of vitamin D deficiency on globular substances in mice. Otol Neurotol. (2021) 42:e1313–7. 10.1097/MAO.0000000000003229 34121084

[11] Sanyelbhaa H Sanyelbhaa A. Vestibular-evoked myogenic potentials and subjective visual vertical testing in patients with vitamin D deficiency/insufficiency. Eur Arch Otorhinolaryngol. (2015) 272:3233–9. 10.1007/s00405-014-3395-6 25411075

[12] Jeong S Kim J Kim H Choi J Koo J Choi K. Prevention of benign paroxysmal positional vertigo with vit D supplementation: a randomized trial. Neurology. (2020) 95:e1117–25. 10.1212/WNL.0000000000010343 32759193

[13] Sheikhzadeh M Lotfi Y Mousavi A Heidari B Monadi M. Bakhshi. EInfluence of supplemental vitamin D on intensity of benign paroxysmal positional vertigo: a longitudinal clinical study. Caspian J Intern Med. (2016) 7:93–8. 27386060 PMC4913711

[14] Zhang Y Liu B Wang Y Zhou Y Wang R Gong J. Analysis of reliability and validity of the Chinese version of dizziness handicap inventory (DHI) (in Chinese). Chin J Otorhinolaryngol Head Neck Surg. (2015) 50:738–43. 10.3760/cma.j.issn.1673-0860.2015.09.008 26696346

[15] Holick M Binkley N Bischoff-Ferrari H Gordon C Hanley D Heaney R. Endocrine Society. Evaluation, treatment, and prevention of vitamin D deficiency: an Endocrine Society clinical practice guideline. J Clin Endocrinol Metab. (2011) 96:1911–30. 10.1210/jc.2011-0385 21646368

[16] Yetiser S Ince D Gul M. An analysis of vestibular evoked myogenic potentials in patients with benign paroxysmal positional vertigo. Ann Otol Rhinol Laryngol. (2014) 123:686–95. 10.1177/0003489414532778 24789801

[17] Oh K Suh K Lee Y Lee S Chang M Mun S. Clinical utility of cervical vestibular-evoked myogenic potentials in predicting residual dizziness after benign paroxysmal positional vertigo. Clin Neurophysiol. (2019) 130:95–100. 10.1016/j.clinph.2018.11.005 30497047

[18] Seo T Saka N Ohta S. Sakagami M. Detection of utricular dysfunction using ocular vestibular evoked myogenic potential in patients with benign paroxysmal positional vertigo. Neurosci Lett. (2013) 550:12–6. 10.1016/j.neulet.2013.06.041 23827225

[19] Lundberg YW Xu Y Thiessen K. Kramer K. Mechanisms of otoconia and otolith development. Dev Dyn. (2015) 244:239–53. 10.1002/dvdy.24195 25255879 PMC4482761

[20] Zucca G Valli S Valli P Perin P Mira E. Why do benign paroxysmal positional vertigo episodes recover spontaneously? J Vestib Res. (1998) 8:325–9. 10.3233/VES-1998-8404 9652482

[21] Yang H Zhao X Xu Y Wang L He Q. Lundberg YW. Matrix recruitment and calcium sequestration for spatial specific otoconia development. PLoS ONE. (2011) 6:e20498. 10.1371/journal.pone.0020498 21655225 PMC3105080

[22] Rabbitt RD Holman HA. ATP and ACh evoked calcium transients in the neonatal mouse cochlear and vestibular sensory epithelia. Front Neurosci. (2021) 15:710076. 10.3389/fnins.2021.710076 34566562 PMC8455828

[23] Yamauchi D Nakaya K Raveendran N Harbidge D Singh R Wangemann P. Expression of epithelial calcium transport system in rat cochlea and vestibular labyrinth. BMC Physiol. (2010) 10:1. 10.1186/1472-6793-10-1 20113508 PMC2825184

[24] Hoseinabadi R Pourbakht A Yazdani N Kouhi A Kamali M. The effects of abnormality of cVEMP and oVEMP on rehabilitation outcomes in patients with idiopathic benign paroxysmal positional vertigo. Eur Arch Otorhinolaryngol. (2016) 273:643–8. 10.1007/s00405-015-3612-y 25825004

[25] Faralli M Lapenna R Giommetti G Pellegrino C Ricci G. Residual dizziness after the first BPPV episode: role of otolithic function and of a delayed diagnosis. Eur Arch Otorhinolaryngol. (2016) 273:3157–65. 10.1007/s00405-016-3947-z 26926693

[26] Seo T Shiraishi K Kobayashi T Mutsukazu K Fujita T Saito K. Residual dizziness after successful treatment of idiopathic benign paroxysmal positional vertigo originates from persistent utricular dysfunction. Acta Otolaryngol. (2017) 137:1149–52. 10.1080/00016489.2017.1347824 28681630

[27] Kim E Oh S Kim J Yang T Yang S. Persistent otolith dysfunction even after successful repositioning in benign paroxysmal positional vertigo. J Neurol Sci. (2015) 358:287–93. 10.1016/j.jns.2015.09.012 26371697

[28] von Brevern M Schmidt T Schonfeld U Lempert T Clarke AH. Utricular dysfunction in patients with benign paroxysmal positional vertigo. Otol Neurotol. (2006) 27:92–6. 10.1097/01.mao.0000187238.56583.9b 16371853

[29] Suh K Oh S Chae H Lee S Chang M Mun SK. Can osteopenia induce residual dizziness after treatment of benign paroxysmal positional vertigo? Otol Neurotol. (2020) 41:e603–e6. 10.1097/MAO.0000000000002586 32068691

[30] Jiang X He L Gai Y Jia C Li W Hu S. Risk factors for residual dizziness in patients successfully treated for unilateral benign posterior semicircular canal paroxysmal positional vertigo. J Int Med Res. (2020) 48:300060520973093. 10.1177/0300060520973093 33296610 PMC7731704

[31] Tomoda K Motoki N Harada N Iwai H Yamashita T. Effect of histamine on intracellular Ca^2+^ concentration in guinea pig isolated vestibular hair cells. Acta Otolaryngo. (1997) 528:37–40. 9288234

[32] Wan T Yu Y Zhao X Tang P Gong Y. Efficiency of betahistine plus cognitive behavioural therapy on residual dizziniess after successful canalith repositioning procedure for benign paroxysmal positional vertigo. Neuropsychiatr Dis Treat. (2018) 14:2965–71. 10.2147/NDT.S182809 30464481 PMC6223332

